# Supplementary material for: Effects of essential tremor on longevity and mortality rates in families
Source: PLoS One. 2025 Apr 7;20(4):e0320422. doi: 10.1371/journal.pone.0320422 (PMC11975089; doi:10.1371/journal.pone.0320422)
Supplement: S1 File — This file contains S1-S3 Figs and S2 and S3 Tables. (PDF) [file pone.0320422.s002.pdf]

Supplementary Material

Effects of essential tremor on longevity and mortality rates in families

Onur Emre Onat[1,2], Faruk Ustunel[1,3], Cem Akbostanci[4], Kivilcim E. Doganyigit-Revino[5], Merve Sen[6], Emre Can Gunaydin[2], Kaya Bilguvar[7,8], Muhittin Cenk Akbostanci[9]

[1] Beykoz Institute of Life Sciences and Biotechnology, Bezmialem Vakıf University, Istanbul, Turkey

[2] Department of Biotechnology, Institute of Health Sciences, Bezmialem Vakıf University, Istanbul, Turkey

[3] Department of Drug Discovery and Development, Institute of Health Sciences, Bezmialem Vakıf University, Istanbul, Turkey

[4] School of Psychology, Washington State University, WA, USA.

[5] Department of Biomolecular Engineering, School of Engineering, University of California Santa Cruz, CA, USA.

[6] Centre for Ophthalmology, Institute for Ophthalmic Research, Universitatsklinikum Tübingen, Tübingen, Germany.

[7] Department of Medical Genetics, School of Medicine, Acıbadem Mehmet Ali Aydınlar University, Istanbul, Turkey

[8] Departments of Neurosurgery and Genetics, School of Medicine, Yale University, New Haven, CT, USA

[9] Department of Neurology, Faculty of Medicine, Ankara University, Ankara, Turkey.

This PDF file includes:

Supplementary Figures 1 to 3 (Pages 2-4)

Supplementary Tables 2 to 3 (Pages 5-6)

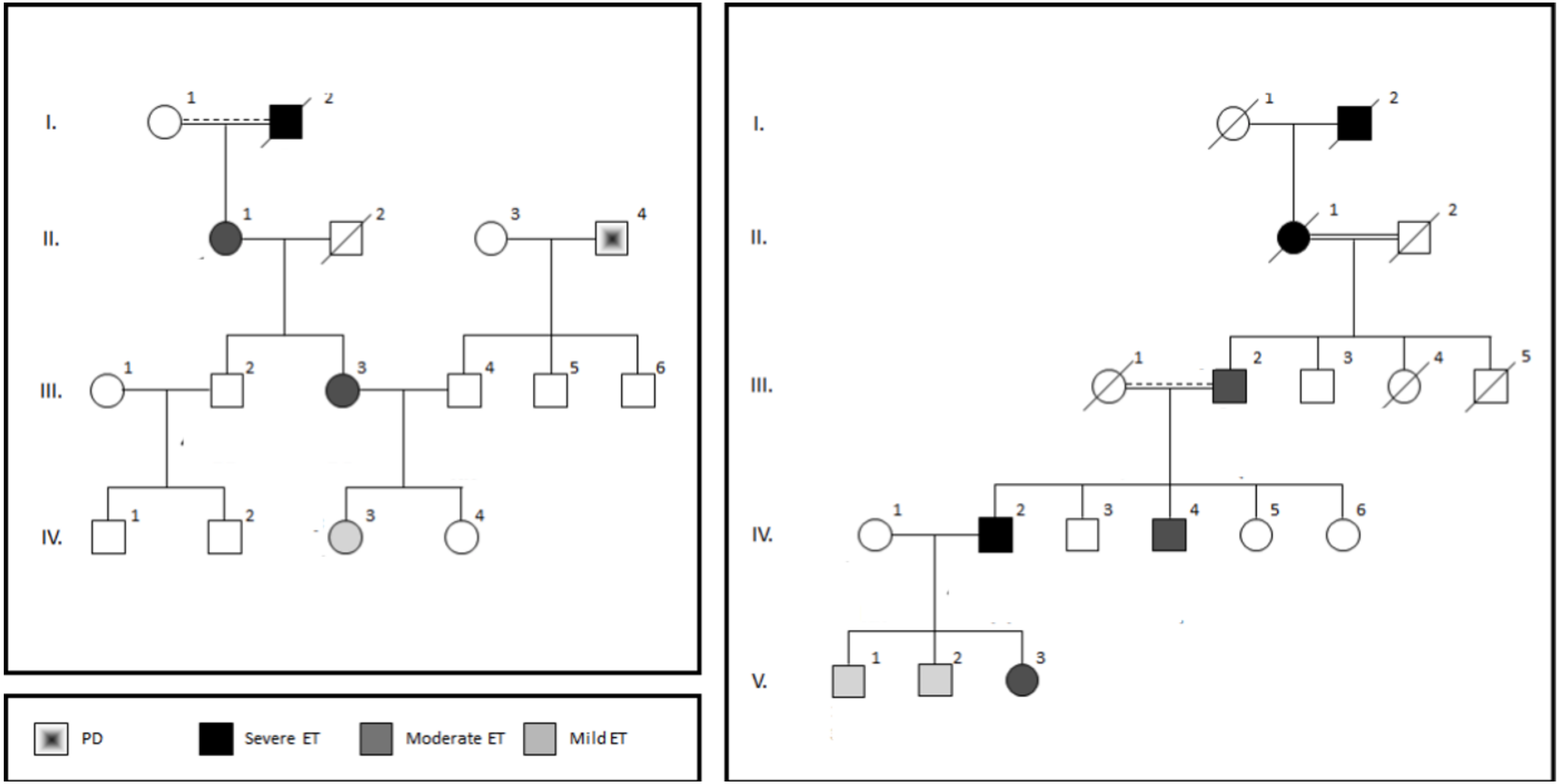

Figure S1: Pedigrees of two families. The age at onset of tremor for affected individuals and the current ages are indicated in this order under the symbols. Probands are indicated with asterisks.

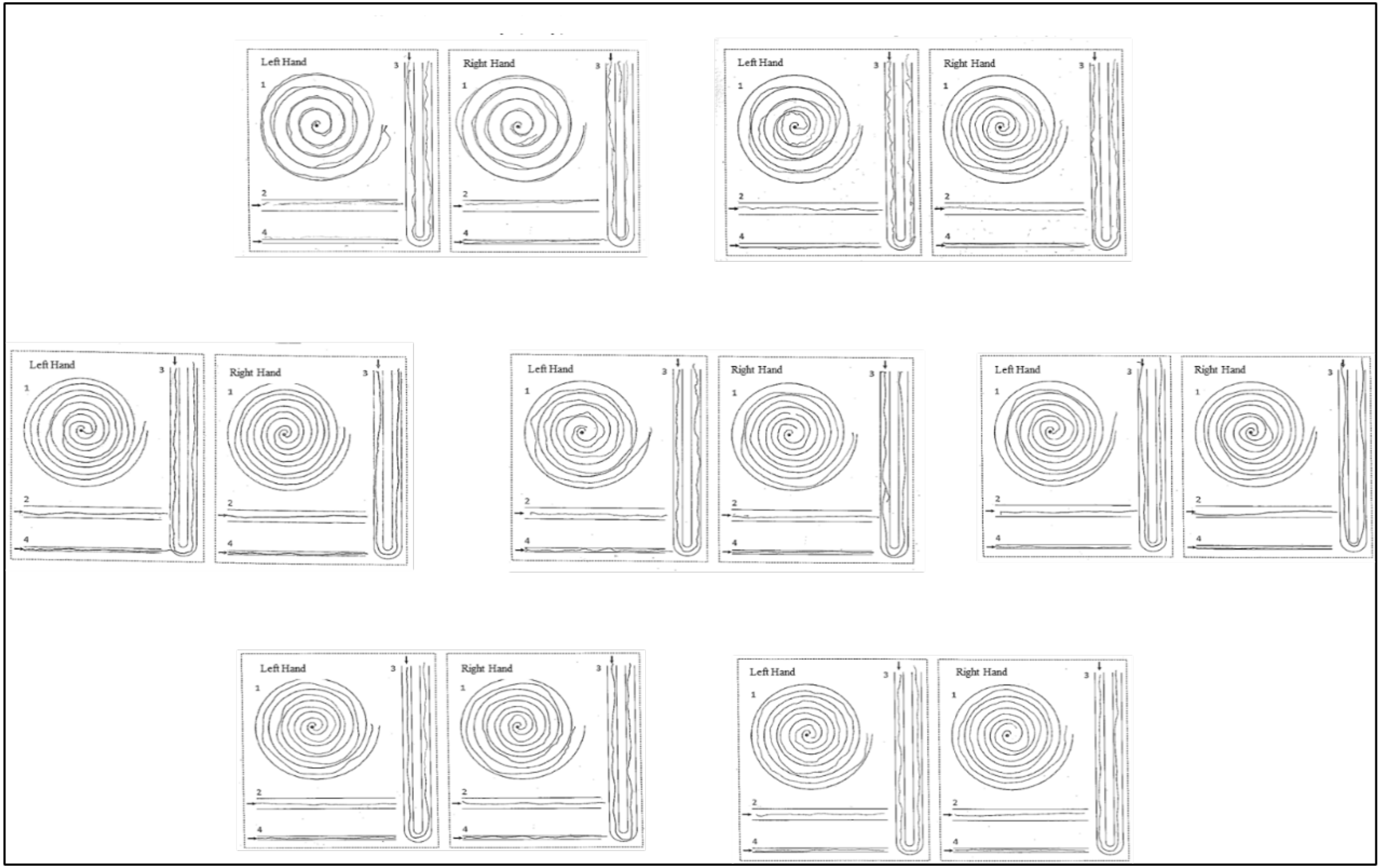

Figure S2: Archimedes spiral drawings of the family (left). Family members, including affected and unaffected individuals.

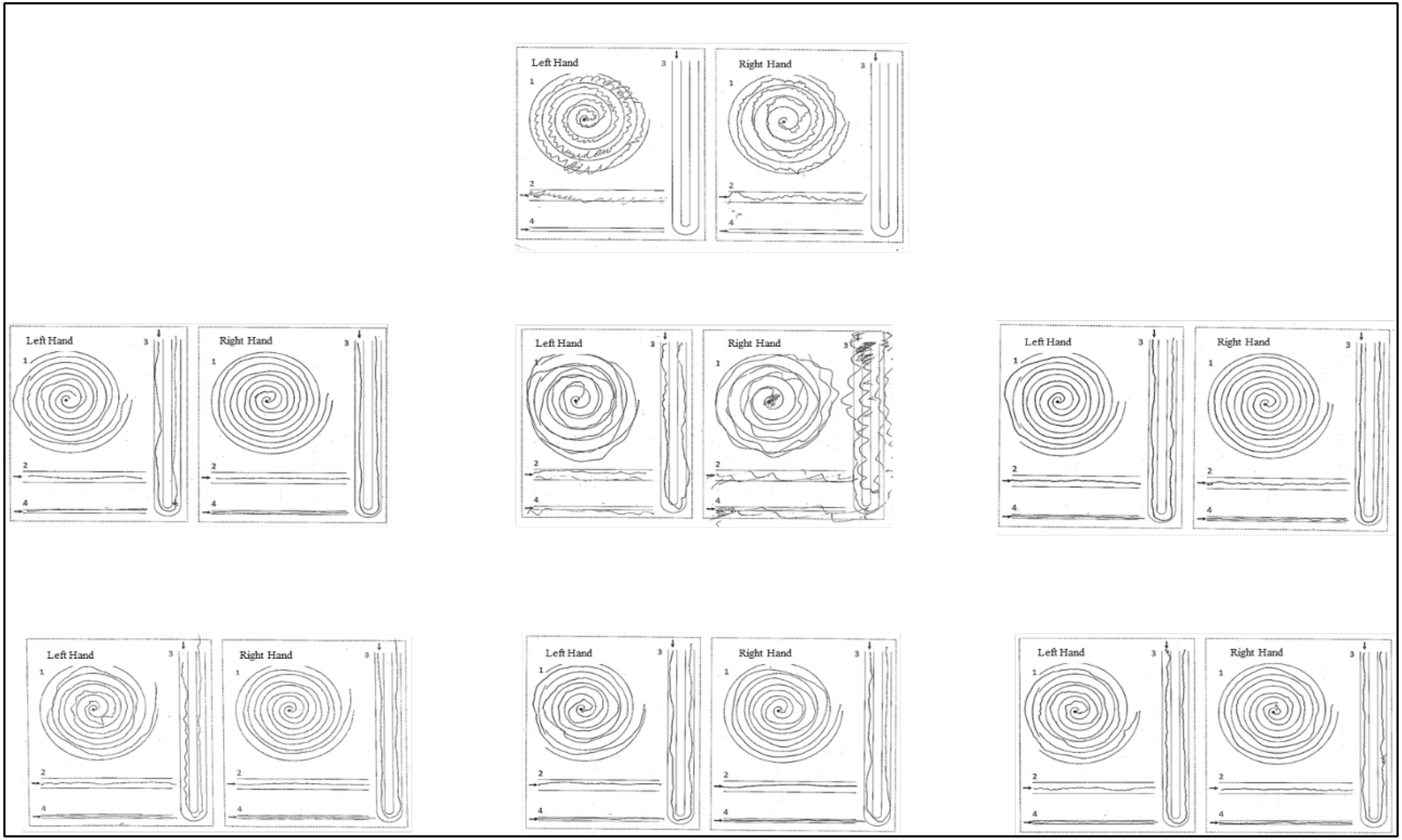

Figure S3: Archimedes spiral drawings of the family (right). Family members, including affected and unaffected individuals.

Table S2: Clinical table of the two families. For tremor +, low amplitude or barely perceivable tremor; ++, moderate amplitude tremor (1-2 cm); +++, large amplitude, severe tremor (>2 cm). For Archimedes' spiral test, bradykinesia, rigidity, postural instability, and hypomimia: +, mild; ++, moderate; +++, severe.

Clinical Severity	Age of Onset	Hypomimia	Tremor											Bradikinesia		Rigidity		Postural Instability	Freezing	Dominant Hand	Archimedes Spiral Test	
			Resting		Postural		Kinetic		Intentional		Head	Chin	Voice									
			Right	Left	Right	Left	Right	Left	Right	Left				Right	Left	Right	Left				Right	Left
Moderate	20	-	++	++	++	++	++	++	++	++	No	No	No	-	-	-	-	-	No	Right	+	+
-	-	-	-	-	-	+	-	-	-	-	No	No	No	-	-	-	-	-	No	Right	-	-
Moderate	19	-	++	++	++	++	+	+	++	++	No	No	No	-	-	-	-	-	No	Right	+	+
-	-	-	-	-	-	-	-	-	-	+	No	No	No	-	-	-	-	-	No	Right	-	-
Mild	5	-	++	+	+	+	+	+	+	+	No	No	No	-	-	-	-	-	No	Left	+	+
-	-	-	-	-	-	-	-	-	-	-	No	No	No	-	-	-	-	-	No	Right	-	-
Severe	8	-	++	++	++	++	++	++	++	++	No	No	No	+	+	-	-	-	No	Right	+	+
-	-	-	-	-	-	-	-	-	-	-	No	No	No	-	-	-	-	+	No	Right	-	-
Severe	8	+	++	-	++	++	++	++	+	+	No	Yes	Yes	+	-	+	+	-	No	Right	++	+++
Moderate	<20	-	+	-	++	+	++	+	+	+	Yes	No	Yes	-	-	+	-	-	No	Right	+	+
Mild	15	-	-	-	-	-	-	+	-	+	No	No	No	-	-	-	-	-	No	Right	+	+
Mild	8	-	+	-	++	+	+	+	+	+	No	No	No	+	-	-	-	-	No	Right	-	-
Moderate	<17	-	+	-	++	++	++	++	-	-	No	No	Yes	-	-	-	-	-	No	Right	+	+

Table S3. **Univariate and multivariate Cox proportional hazards models for mortality.** Univariate unadjusted Cox Models: Patients with ET vs non-ET family members in all cohort and elderly cohort; ET male vs female patients; and non-ET male vs female family members Multivariate Cox model that adjusted for baseline age, disease status, and gender: Males with vs without ET and females with vs without ET. All groups except non-ET male vs female family members are significantly different at P < 0.0001.

Comparison of Survival Curves	Median A	Median B	Log-rank Chi^2	df	P	Summary	Covariate	HR	95% CI
All [ET(+) vs ET(-)]	85	77	100.59	1	<0.0001	****		0.44	0.37 to 0..52
Elderly [ET(+) vs ET(-)]	85	78	65.68	1	<0.0001	****		0.48	-0.91 to -0.54
ET+ [Female vs Male]	85	85	3.73	1	0.053	ns		1.28	1.00 to 1.63
ET- [Female vs Male]	79	73	16.91	1	<0.0001	****		1.57	1.26 to 1.95
Male [ET(+) vs ET(-)]	85	73	68.22	1	<0.0001	****	ET(+)	0.66	0.47 to 0.93
							ET(-)	1.51	1.08 to 2.13
Female [ET(+) vs ET(-)]	85	79	32.02	1	<0.0001	****	ET(+)	0.73	0.52 to 1.02
							ET(-)	1.37	0.98 to 1.93
