# Supplementary material for: Effects of essential tremor on longevity and mortality rates in families
Source: PLoS One. 2025 Apr 7;20(4):e0320422. doi: 10.1371/journal.pone.0320422 (PMC11975089; doi:10.1371/journal.pone.0320422)
Supplement: Attachment — Submitted filename: response_to_reviewers.docx [file pone.0320422.s005.docx]

**Manuscript PONE-D-24-19093R1**

Responses to the Reviewers' comments:

**Reviewer #2:** The aim of this study is to analyze the effects of essential tremor on longevity and mortality rates in families. It is very interesting in all parts. I suggest to revise the language.

***Author Response:*** We sincerely thank the reviewer for their positive feedback and for highlighting the importance of our study. In response to the suggestion to revise the language, we have carefully reviewed and refined the manuscript to enhance its clarity and readability. Additionally, we have incorporated new content and reorganized key sections to improve the overall narrative and ensure the study’s objectives and findings are presented more effectively.

**Reviewer #3:** In this study Onur Emre Onat et al. investigated the potential link between Essential Tremor (ET) and longevity. They included 1,493 participants from 145 families, divided into ET-positive (ET+) and ET-negative (ET-) groups. The analysis aimed to clarify whether ET status is associated with lifespan. Results showed that the median age at death for deceased ET+ individuals was 80 years, compared to 70 years for ET- individuals. Living ET+ individuals had a median age of 63 years, while ET- individuals had a median age of 60 years. Survival analysis revealed a significantly longer lifespan for the ET+ group, with a hazard ratio of 0.44, indicating a lower mortality risk in ET+ patients, particularly among males.

I believe the study presents several methodological weaknesses that undermine the reliability and validity of its conclusions:

We are sincerely grateful for the time and effort you have dedicated to evaluating our work. In response to your comments, we have carefully reviewed and revised the manuscript once again, addressing your concerns to the best of our ability. Specifically, we have addressed issues related to potential confounding factors and study design limitations. Furthermore, we have included new content and reorganized key sections to enhance the overall narrative. Below, we provide a detailed, point-by-point response to your feedback. We hope these revisions comprehensively address your concerns and significantly strengthen the manuscript.

Specific comments:

- The study relies heavily on retrospective data for deceased patients, reconstructed through family trees. This approach introduces substantial recall bias, particularly regarding the accuracy of death diagnoses, age at death, and the presence of tremor. The reliance on family-reported data significantly undermines the validity and reliability of these key

***Author Response:*** Study designs for analyzing aging and/or longevity in essential tremor cases include both retrospective and prospective models. Examples from the literature include:

1. Retrospective Design: One notable retrospective study is based on medical record review utilizing the records linkage system of the Rochester Epidemiology Project in the United States. This study encompassed individuals of all age groups, providing a comprehensive analysis of essential tremor cases across the lifespan (1).

2. Prospective Design: A prominent example of a prospective, population-based study is the NEDICES study in Spain, which focused on individuals aged 65 and older. This study employed a survey-based approach involving family members of elderly individuals. Death was systematically ascertained through data collection on age at death and cause of death from healthcare providers and family members (2).

3.Family-Based Longevity Study: Another approach is a family-based longevity study that evaluated relatives of patients with Parkinson’s disease, essential tremor, and control subjects from the same families. This study collected information on age at death and disease status using a similar survey-based approach with input from family members (3).

As noted in our previous response and discussed in the manuscript, we acknowledge that collecting death information from family members is a limitation of our study. Efforts to access original death certificates were hindered by complex bureaucratic processes and, in some cases, the reluctance of family members to engage in these procedures. We discussed this issue in the Limitations and Challenges part of the Discussion section.

Revised Text (Page 16, Lines 575–592):

Additionally, the lack of detailed cause-of-death information, particularly for older individuals or those from rural areas, limits our ability to explore specific mortality patterns in depth. Deaths categorized as “natural causes” often lack specificity, potentially obscuring critical insights into comorbidities and other factors influencing survival. Access to more precise cause-of-death data, such as death certificates or autopsy reports, would provide valuable insights into whether specific comorbidities or conditions, such as cardiovascular disease or neurodegenerative disorders, are common in ET patients compared to the general population. Furthermore, our exclusion of individuals who died from non-age-related causes (e.g., accidents or suicides) may introduce bias by narrowing the study population to those with age-related mortality. While this approach was necessary to focus on longevity, it may limit the generalizability of our findings. We cannot entirely rule out this possibility, however our analysis of individuals aged 60 and older still demonstrated a significant increase in survival times for ET patients (Log-rank *p* = 5.29 × 10^-16^ (Fig 3B)), suggesting that the longevity benefit associated with ET is robust even among older individuals. Additionally, our study did not account for certain lifestyle factors, such as diet, physical activity, and alcohol consumption, which may influence both ET development and longevity. Further studies that incorporate detailed data on these factors would help to clarify their potential impact.

That said, we emphasize the robustness of our data collection methods. Recruitment occurred during family appointments where multiple members were present, allowing for coordinated and cross-verified responses. Pedigree drawings and documentation of living and deceased first- and second-degree relatives, as well as tremor reports, were completed collaboratively and cross-checked in real time. These efforts were designed to enhance the specificity and accuracy of the data.

Revised Text (Page 5, Lines 144–152):

The accuracy of the pedigree information was critical for genetic analysis, and multiple checks were incorporated into the data collection process to ensure reliability and completeness. Recruitment of family members was coordinated during follow-up appointments, and efforts were made to have as many family members present as possible during these visits. This strategy facilitated the construction of robust pedigree charts, as family members could cross-verify information, minimizing discrepancies. During these appointments, family members collaborated to name and list all living and deceased first- and second-degree relatives, and they jointly reported on the presence of tremors in the family. This cross-referencing process ensured high specificity and accuracy in the familial data collected.

While a longitudinal prospective study would be ideal, it is not feasible given the retrospective nature of our research and the high proportion of deceased individuals. To address this limitation, we tested our data collection methods using 10 well-characterized multigenerational families, encompassing both affected and unaffected members. Despite the small sample size, we confirmed our observations in all families, with significant differences evident in 3 of the 10 families. Furthermore, we compared the survival status of ET patients with and without clinical assessments and found no significant difference in age at death between these groups (Figure 2E).

Revised Text (Page 16, Lines 565–575):

Despite its strengths, our study has limitations that warrant consideration. First, 35.6% of ET+ cases and 44.3% of ET- individuals were deceased family members, and 22 individuals had died after the baseline evaluation. For many of these individuals, assessments were based on family-reported histories rather than direct clinical evaluations, which introduces the potential for recall bias and misclassification of ET status. To mitigate this, we compared survival data between ET patients with and without clinical assessments, finding a significant difference in age at death between the two groups (U = 2.99 × 103, p = 2 × 10-3) (Fig 2E). Furthermore, we examined 10 phenotypically well-characterized multigenerational families, finding that in 3 of the 10 families, ET patients lived longer than their non-ET relatives. This finding suggests that genetic factors contributing to ET may also play a role in influencing longevity.

References:

1. Rajput AH, Offord KP, Beard CM, Kurland LT: Essential tremor in Rochester, Minnesota: a 45-year study. J Neurol Neurosurg Psychiatry 1984; 47: 466–470.
2. Benito-Leon J, Bermejo-Pareja F, Louis ED: Incidence of essential tremor in three elderly populations of central Spain. Neurology 2005; 64: 1721–1725.
3. Jankovic J, Beach J, Schwartz K, Contant C. Tremor and longevity in relatives of patients with Parkinson's disease, essential tremor, and control subjects. Neurology. 1995 Apr;45(4):645-8.

- The inclusion of participants from the same families raises concerns about genetic and environmental confounding. Family-based sampling does not control for hereditary factors that could influence both tremor and longevity. The diversity in socio-economic backgrounds within the sample is insufficient to ensure the external validity of the results, as it does not reflect the heterogeneity of the general population.

***Author Response:*** We appreciate the reviewer’s concerns regarding genetic and environmental confounding and the external validity of our findings. As noted in the literature (1-4), family-based sampling is a powerful approach for jointly studying genetic and environmental factors, offering unique insights that may not be captured in population-based studies. While family-based cohorts inherently focus on hereditary aspects, they also allow for robust intra-family comparisons under shared environmental conditions (5-14). We believe this design is an advantage for investigating the genetic epidemiology of essential tremor (ET).

Our cohort predominantly consists of families residing in small villages, where members share similar socioeconomic conditions, dietary habits, and microbial exposures. This homogeneity helps mitigate potential confounding factors across different families. To further address potential biases, we conducted separate analyses within each family (Fig. 4) and compared these results to pooled analyses across all families (Fig. 1). Both approaches yielded consistent findings, supporting the validity of our conclusions.

However, we agree that the generalizability of findings from family-based cohorts to the broader population must be interpreted cautiously. To address this limitation, we have emphasized in the manuscript the importance of complementing family-based studies with population-based research. Our expanded discussion highlights the need for additional studies to explore the interplay between genetic and environmental factors in ET and its potential impact on longevity. Relevant revisions to the manuscript are as follows:

Revised Text (Page 3, Lines 73–103):

While neurodegenerative diseases are generally associated with shortened life expectancy, the impact of ET on longevity remains unclear [[17–20]](https://www.zotero.org/google-docs/?zGXi7S). Early observational studies such as that by Minor, suggested a potential survival advantage for individuals with ET [[17,18]](https://www.zotero.org/google-docs/?WG0dw1), yet subsequent research has produced contradictory findings. For example, a longitudinal retrospective study conducted in 1984 by Rajput *et al.*, on 266 ET patients, found no evidence of increased longevity. However, this study was limited by its relatively young cohort and insufficient follow-up into advanced age [[19]](https://www.zotero.org/google-docs/?eMv2tS). Conversely, a more comprehensive study by Jankovic *et al.* in 1995 examined 201 ET patients and compared them to 465 age-matched controls in a longitudinal prospective design. Their results indicated significantly increased longevity among ET patients with a relative risk [RR] of 1.59, (*p* = 0.01) [[20]](https://www.zotero.org/google-docs/?tipD7L). More recently, a population-based prospective study conducted in three villages in central Spain in 2007 by Louis *et al.* suggested that ET may even increase mortality risk under certain conditions [[21]](https://www.zotero.org/google-docs/?Q16Jlw). These conflicting results highlight the need for further investigation into the complex relationship between ET and lifespan.

To address these uncertainties further investigation into the relationship between ET and mortality is crucial. There is a clear need for more rigorous, family-based studies to investigate how ET might influence survival rates, particularly by accounting for genetic factors and controlling for confounding variables such as diet and socioeconomic status. Additionally, there is a lack of data exploring gender-specific differences in mortality and how such differences could contribute to our understanding of ET’s impact on longevity. In our comprehensive family-based retrospective study, we aimed to address this gap by comparing mortality risk in ET patients to their family members. By comparing survival outcomes among individuals with and without ET, and accounting for potential confounding factors, this work seeks to provide new insights into the potential link between ET and extended lifespan. In this study, we examined the pedigrees of 145 probands and tested the association of ET with longevity in 1,493 subjects. Our findings suggest that ET could indeed be associated with longer life expectancy, offering an intriguing avenue for further research into the underlying mechanisms that might confer this potential longevity advantage. These findings, if confirmed in large-scale longitudinal studies and biological investigations, could have significant implications for public health, particularly in understanding the complex interplay between ET, aging and survival.

Revised Text (Page 13-14, Lines 460–481):

Genetic Contributions to Longevity in ET. Genetic factors are likely to contribute significantly to the observed association between ET and increased lifespan. Several loci associated with ET, including those implicated in mitochondrial function and oxidative stress response, could influence both the development of tremor and the broader biological pathways governing aging. These genetic variations may provide protection against age-related diseases, such as cardiovascular conditions or metabolic syndromes, which are leading causes of mortality in the general population. Our family-based study design offers unique insights into the potential genetic basis of ET-related longevity. By comparing ET-positive (ET+) individuals to their ET-negative (ET-) relatives, we were able to control for many environmental and socioeconomic variables. By studying family members from the same households and communities, we sought to minimize these confounding variables. However, this design also introduces the potential for selection bias, as the families we studied may not represent the broader population. The genetic uniqueness of our cohort is underscored by an inbreeding coefficient of 36.5%, calculated from exome sequencing data, indicating a higher degree of genetic relatedness within these families than would be typical in the general population. The consistent trend of longer lifespans among ET+ individuals within the same families underscores the possibility that genetic factors influencing ET may also contribute to longevity. Our findings provide important insights into the relationship between ET and mortality. We found that the median age of death for the ET+ group was significantly higher than for ET- individuals (80 years vs. 70 years), suggesting that ET might be associated with increased longevity. This was further supported by survival analysis, which revealed a statistically significant higher risk of mortality in ET- individuals compared to those with ET.

References

1. Hopper, J. L., Bishop, D. T., & Easton, D. F. (2005). Population-based family studies in genetic epidemiology. Lancet (London, England), 366(9494), 1397–1406.
2. Lawlor, Deborah A., and Gita D. Mishra (eds), Family matters: Designing, analysing and understanding family based studies in life course epidemiology (Oxford, 2009; online edn, Oxford Academic, 1 Sept. 2009),
3. Stein CM, Hall NB, Malone LL, Mupere E. The household contact study design for genetic epidemiological studies of infectious diseases. Front Genet. 2013 Apr 30;4:61.
4. Morris N, Elston RC, Barnholtz-Sloan JS, Sun X. Novel approaches to the analysis of family data in genetic epidemiology. Front Genet. 2015 Feb 6;6:27.
5. Estus, J. L., Family Investigation of Nephropathy Diabetes Research Group, and Fardo, D. W. (2013). Combining genetic association study designs: a GWAS case study. Front. Genet. 4:186.
6. Huang, Y., Thomas, A., and Vieland, V. J. (2013). Employing MCMC under the PPL framework to analyze sequence data in large pedigrees. Front. Genet. 4:59.
7. Lutz, S. M., Hokanson, J. E., and Lange, C. (2014). An alternative hypothesis testing strategy for secondary phenotype data in case-control genetic association studies. Front. Genet. 5:188.
8. Lutz, S. M., Vansteelandt, S., and Lange, C. (2013). Testing for direct genetic effects using a screening step in family-based association studies. Front. Genet. 4:243.
9. Nelson, Q., Agarwal, N., Stephenson, R., and Cannon-Albright, L. A. (2013). A population-based analysis of clustering identifies a strong genetic contribution to lethal prostate cancer. Front. Genet. 4:152.
10. Song, Y. E., and Elston, R. C. (2013a). The null distribution of likelihood-ratio statistics in the conditional-logistic linkage model. Front. Genet. 4:244.
11. Song, Y. E., and Elston, R. C. (2013b). PedWiz: a web-based tool for pedigree informatics. Front. Genet. 4:189.
12. Stein, C. M., Hall, N. B., Malone, L. L., and Mupere, E. (2013). The household contact study design for genetic epidemiological studies of infectious diseases. Front. Genet. 4:61.
13. Uemoto, Y., Pong-Wong, R., Navarro, P., Vitart, V., Hayward, C., Wilson, J. F., et al. (2013). The power of regional heritability analysis for rare and common variant detection: simulations and application to eye biometrical traits. Front. Genet. 4:232.
14. Wang, H. Z., Qin, H. D., Guo, W., Samuels, J., and Shugart, Y. Y. (2013). New insights into the genetic mechanism of IQ in autism spectrum disorders. Front. Genet. 4:195.

- The study does not sufficiently address the potential inclusion of patients exhibiting parkinsonian features within the ET cohort. Without clear diagnostic criteria distinguishing ET or ET plus from Parkinson's disease (PD), there is a risk of misclassification, which could substantially alter the study's outcomes. Moreover, the absence of a comprehensive follow-up to confirm the absence of PD or other neurodegenerative conditions raises further concerns about diagnostic accuracy.

***Author Response:*** We thank you for highlighting the potential for diagnostic misclassification between essential tremor (ET) and Parkinson’s disease (PD). To address this concern, we employed rigorous diagnostic criteria to ensure accurate classification. Specifically, we utilized the Washington Heights-Inwood Genetic Study of Essential Tremor (WHIGET) criteria and the Consensus Statement of the Movement Disorder Society on Tremor (MDS) to diagnose ET. Furthermore, all participants underwent systematic evaluation for bradykinesia, muscular rigidity, and postural instability using the UK Parkinson Disease Society Brain Bank diagnostic criteria. These protocols allowed us to distinguish between ET, ET plus, and PD with a high degree of accuracy.

Revised Text (Page 4, Lines 123–143):

Patients admitted to the Neurology Department of Ankara University Medical School Hospital with the complaint of tremor were invited to participate in the genetic study if they reported having multiple affected and unaffected individuals in their families. Each proband underwent a comprehensive 90-minute baseline medical interview. If patients exhibited neurological symptoms, such as cognitive impairment, bradykinesia, or tremor, they were referred for detailed and follow-up neurological evaluations. We preferentially recruited familial early-onset ET cases, particularly those classified as “pure ET”. Clinical information parameters included: a. type of tremor, b. presence or absence of Parkinson’s disease, c. presence or absence of dystonia, d. response to treatment, e. history of drug use (e.g., lithium), f. substance use (e.g., alcohol or drug addiction), g. exposure to chemicals or toxins. Exclusion criteria were strictly applied to enhance the homogeneity of the study group and minimize confounding variables. We excluded individuals diagnosed with dementia, Parkinson's disease, or any other neurodegenerative disease, as well as those with known vascular, demyelinating, or structural brain lesions (e.g., masses). Additionally, individuals taking medications known to induce action tremor or those with severe hyperthyroidism were also excluded. However, recognizing the association between ET and a heightened risk of Parkinson’s disease, patients with ET who exhibited overlapping parkinsonism features (e.g., bradykinesia, rigidity, and non-motor symptoms) were not excluded from the study. Demographic information for each proband was collected during the initial interview. Probands were asked to provide detailed information about all living or deceased first- and second-degree relatives and indicate whether any relatives had tremors.

This comprehensive screening minimized the risk of misclassification, ensuring that overlapping parkinsonian features were appropriately identified and accounted for. While we acknowledge the inherent challenges in differentiating ET from PD in some cases, the use of these well-established criteria reflects best practices in the field and strengthens the validity of our findings.

Revised Text (Page 5, Lines 153–177):

Ankara University Medical School physicians initially evaluated ET patients, and their family members based on clinical and functional performance tests. All clinical examinations were performed according to the Helsinki Declaration [[25]](https://www.zotero.org/google-docs/?vWRgwm). Each proband and family member recruited for the study was assigned a unique DNA code and subjected to comprehensive general physical and detailed neurological examination. The diagnostic criteria for ET were aligned with the Washington Heights-Inwood Genetic Study of Essential Tremor (WHIGET) [[26]](https://www.zotero.org/google-docs/?MWjZ7c) and the Consensus Statement of the Movement Disorder Society on Tremor (MDS) [[27]](https://www.zotero.org/google-docs/?pOxBhy). The severity of resting and postural tremors for each participant was graded from 0 to +3: 0, no visible tremors; +1, low-amplitude tremor; +2, moderate-amplitude tremor; and +3, high-amplitude tremor. To assess kinetic tremor, participants were examined while performing four different tasks: finger-to-nose movement, pouring water, drinking water, and drawing. ET patients were also evaluated for bradykinesia, muscular rigidity, and postural instability to assess the presence of Parkinson’s disease or dystonia according to the UK Parkinson Disease Society Brain Bank diagnostic criteria [[3,28]](https://www.zotero.org/google-docs/?umGije). This careful screening ensured that patients with overlapping parkinsonian features were appropriately identified and accounted for in the study.

During the clinical assessments, a trained research assistant collected demographic data (e.g., age, gender, race, and education level), as well as detailed information about the age of symptom onset, family history of ET, family history of Parkinson’s disease, medication usage, and pedigree information. Enrollment of probands and their family members began in 2010 and was completed in 2019. Over this period, 161 families were initially recruited for the study; however, 16 families were excluded due to the inability to perform follow-up analysis resulting from a loss of contact. This exclusion ensured that the final dataset consisted only of participants with confirmed ET or individuals closely related to ET patients, with a final cohort drawn from 145 families **(S1 Table)**.

For deceased ET cases, direct clinical evaluation was not possible. However, ET is a highly heritable condition, with familial inheritance observed in 50–70% of cases (1) and high concordance rates of 69–93% in monozygotic twins (2). Given this strong genetic basis, we believe the use of family histories to evaluate deceased individuals minimizes the likelihood of misclassification.

Revised Text (Page 17, Lines 616–624):

**Conclusion.** In summary, our study adds to the limited body of literature on ET and mortality by suggesting that ET may be associated with increased lifespan, particularly in male patients and those with late-onset disease. However, our findings should be interpreted with caution due to the study’s limitations, including the reliance on self-reported family histories and the lack of detailed cause-of-death data. Future research should aim to collect more comprehensive biological, genetic, and lifestyle data, as well as to include larger and more diverse cohorts, to validate and extend these findings. Clarifying the mechanisms underlying the relationship between ET and longevity could yield important insights into both ET pathophysiology and the broader biological processes that contribute to healthy aging.

References

1. Louis ED, Ferreira JJ. How common is the most common adult movement disorder? Update on the worldwide prevalence of essential tremor. Mov Disord. 2010;25:534-541.
2. Lorenz D, Frederiksen H, Moises H, Kopper F, Deuschl G, Christensen K. High concordance for essential tremor in monozygotic twins of old age. Neurology. 2004;62:208-211.

- The results, which suggest enhanced longevity in ET patients, are contrary to the possible understanding of ET as a neurodegenerative condition. The study does not adequately explore the potential biological mechanisms that could explain this surprising association between ET and increased lifespan. Given the neurodegenerative nature of the disorder, the authors should provide a more detailed discussion of the underlying pathophysiology that could account for the observed mortality differences, as this remains an unexplained and significant gap with respect to the current literature.

***Author Response:*** Thank you for your valuable comment regarding the need to explore potential biological mechanisms that could explain the association between ET and enhanced longevity. The observation of enhanced longevity in essential tremor (ET) patients, despite ET’s classification as a neurodegenerative disorder, presents an intriguing paradox, which may be explained by several factors. First, recent studies suggest that ET may not represent a single disorder but rather a spectrum of conditions with varying underlying mechanisms (Louis, 2016). This heterogeneity could mean that certain subtypes of ET exhibit less aggressive neurodegenerative processes, potentially contributing to increased longevity in specific patient populations (Louis & Faust, 2020). The heterogeneity of ET, highlighted in neuroimaging and pathological studies, supports the idea that not all ET cases involve the same degree of neurodegeneration (Louis, 2019). Secondly, it is possible that in some ET patients, compensatory neural mechanisms mitigate the effects of neurodegeneration. This could preserve neurological function and contribute to a longer lifespan. However, further studies are needed to explore this hypothesis in detail, particularly using advanced neuroimaging techniques to assess adaptive neural changes in ET (Benito-León et al., 2013). Third, genetic studies have identified specific loci associated with ET, including LINGO1 and STK32B, which might also influence longevity (Thier et al., 2019). Some of these genetic factors may confer resilience to other age-related conditions, such as cardiovascular or metabolic diseases, which are common causes of mortality in the general population. These protective genetic traits could help explain the observed association between ET and increased lifespan. Fourth, individuals with ET often adopt lifestyle modifications to manage their symptoms, such as engaging in regular physical activity or consuming certain diets. These practices may have additional health benefits that promote longevity. While this hypothesis remains speculative, studies have shown that lifestyle interventions can significantly impact lifespan in other neurological conditions (Benito-León et al., 2009). Lastly, it is important to consider the potential for selection bias in studies reporting enhanced longevity in ET patients. For instance, individuals with milder ET forms who live longer may be more likely to participate in research, which could skew the results (Louis, 2005). Careful design of future studies is essential to address this limitation. Given the surprising nature of these findings, further research is critical. Longitudinal cohort studies with detailed genetic profiling and neuroimaging data could help elucidate the biological mechanisms underlying the association between ET and increased lifespan (Benito-León & Louis, 2006). In response, we have expanded the discussion section to address this gap as in the following:

Revised Text (Page 17, Lines 616–624):

**Interpreting the Longevity Paradox in ET.** The apparent longevity benefit in ET patients raises intriguing questions about the biological and clinical underpinnings of this association. While ET is traditionally considered a neurodegenerative disorder, its classification is evolving. Growing evidence suggests that ET represents a spectrum of conditions rather than a singular disease entity [40]. Subtypes of ET may differ in their underlying mechanisms, ranging from degenerative processes involving the cerebellum to milder, age-related phenomena. This heterogeneity could mean that specific forms of ET are associated with less severe neurodegeneration or even adaptive changes that confer survival advantages [8]. The heterogeneity of ET, highlighted in neuroimaging and pathological studies, supports the idea that not all ET cases involve the same degree of neurodegeneration [8].

Addionally, it is possible that in some ET patients, compensatory neural mechanisms mitigate the effects of neurodegeneration. This could preserve neurological function and contribute to a longer lifespan. However, further studies are needed to explore this hypothesis in detail, particularly using advanced neuroimaging techniques to assess adaptive neural changes in ET [41].

Several studies have identified genetic variations associated with ET that could be linked to pathways involved in aging and longevity. For instance, mutations in genes related to mitochondrial function or oxidative stress response pathways could influence both the development of ET and the lifespan of affected individuals [39,42,43]. Additionally, familial aggregation of ET may suggest the presence of genetic modifiers that influence not only tremor development but also overall health and longevity [7]. Some of these genetic factors may confer resilience to other age-related conditions, such as cardiovascular or metabolic diseases, which are common causes of mortality in the general population. For instance, genetic studies have identified specific loci associated with ET, including LINGO1 and STK32B, which might also influence longevity [7]. These protective genetic traits could help explain the observed association between ET and increased lifespan.

One more plausible explanation for the increased longevity observed in our study could be improved medical compliance among individuals with ET. Chronic conditions such as ET often require regular medical check-ups and adherence to prescribed therapies, which could translate into better management of comorbidities and early detection of other health issues. This is consistent with findings from other studies, which have shown that individuals with chronic diseases tend to seek more frequent medical care and engage in healthier lifestyle practices that contribute to prolonged life. Furthermore, patients with ET may be more likely to adopt preventive measures such as regular exercise or medications, which could contribute to their extended lifespan [[44–46]](https://www.zotero.org/google-docs/?Rjj9kp).

Environmental and lifestyle factors, including diet, physical activity, and alcohol consumption, could also play a role in the longevity of ET patients. Previous research has indicated that moderate alcohol consumption may have neuroprotective effects, potentially alleviating tremor symptoms and improving motor function [[46,47]](https://www.zotero.org/google-docs/?wXnHCx). Exercise, which has been shown to improve cognitive function and overall health, could also have a beneficial effect on individuals with ET, mitigating some of the negative consequences of aging [48,49]. Furthermore, dietary factors such as caloric restriction have been linked to extended lifespan in both animal models and human populations, and it is possible that individuals with ET may adopt diets that reduce inflammation and oxidative stress, both of which contribute to aging [7,43].

Lastly, it is important to consider the potential for selection bias in studies reporting enhanced longevity in ET patients. For instance, individuals with milder ET forms who live longer may be more likely to participate in research, which could skew the results [1]. Careful design of future studies is essential to address this limitation. Given the surprising nature of these findings, further research is critical. Longitudinal cohort studies with detailed genetic profiling and neuroimaging data could help elucidate the biological mechanisms underlying the association between ET and increased lifespan [50].

References:

1. Benito-León, J., & Louis, E. D. (2006). Essential tremor: Emerging views of a common disorder. Nature Clinical Practice Neurology, 2(12), 666-678.
2. Louis, E. D. (2005). Essential tremor. The Lancet Neurology, 4(2), 100-110.
3. Benito-León, J., et al. (2009). Lifestyle factors and risk of essential tremor. Movement Disorders, 24(11), 1773-1778.
4. Louis, E. D., & Faust, P. L. (2020). Essential tremor pathology: Neurodegeneration and its implications. Movement Disorders, 35(8), 1360-1367.
5. Thier, S., Lorenz, D., & Deuschl, G. (2019). Genetics of essential tremor: A review. Tremor and Other Hyperkinetic Movements, 9(1), 1-8.
