# Supplementary material for: Effects of essential tremor on longevity and mortality rates in families
Source: PLoS One. 2025 Apr 7;20(4):e0320422. doi: 10.1371/journal.pone.0320422 (PMC11975089; doi:10.1371/journal.pone.0320422)
Supplement: Attachment — Submitted filename: Response to Reviewers.docx [file pone.0320422.s004.docx]

**Manuscript PONE-D-24-19093: Response to Reviewers**

Dear Dr. Hasan,

Thank you for providing us the opportunity to submit a revised draft of our manuscript titled “Effects of Essential Tremor on Longevity and Mortality Rates in Families” for publication in *PLOS ONE*. We sincerely appreciate the time and effort you and the reviewers have dedicated to evaluating our work. The insightful comments and valuable suggestions we received have greatly contributed to improving the quality of our manuscript.

We have carefully considered all the feedback provided, and we have incorporated most of the reviewers’ suggestions into the revised manuscript. In response to your guidance, we rewrote the entire text and utilized the track changes feature in Word to clearly highlight all modifications. Additionally, we have prepared a clean version of the manuscript for your convenience. Below, we provide a point-by-point response to the reviewers’ comments and concerns in blue font. Please note that all page numbers mentioned correspond to the revised manuscript without tracked changes.

With my best regards,

Onur Emre Onat

**Journal Requirements:**

When submitting your revision, we need you to address these additional requirements.

1. Please ensure that your manuscript meets PLOS ONE's style requirements, including those for file naming. The PLOS ONE style templates can be found at
<https://journals.plos.org/plosone/s/file?id=wjVg/PLOSOne_formatting_sample_main_body.pdf> and
<https://journals.plos.org/plosone/s/file?id=ba62/PLOSOne_formatting_sample_title_authors_affiliations.pdf>

***Author Response:*** We have carefully reviewed the style templates and revised the entire manuscript to fully adhere to the “*PLOS ONE*” formatting guidelines.

2. Thank you for stating the following in the Competing Interests section: "The authors declare that the research was conducted without any commercial or financial relationships that could be construed as a potential conflict of interest.” Please confirm that this does not alter your adherence to all PLOS ONE policies on sharing data and materials, by including the following statement: "This does not alter our adherence to PLOS ONE policies on sharing data and materials.” (as detailed online in our guide for authors <http://journals.plos.org/plosone/s/competing-interests>). If there are restrictions on sharing of data and/or materials, please state these. Please note that we cannot proceed with consideration of your article until this information has been declared. Please include your updated Competing Interests statement in your cover letter; we will change the online submission form on your behalf.

***Author Response:*** We have included the following statement, which was previously categorized under the “Conflict of Interest Statement” section, now updated to “Competing Interests.

Page 17, Lines 611-614

The authors declare that the research was conducted without any commercial or financial relationships that could be construed as a potential conflict of interest. This does not alter our adherence to PLOS ONE policies on sharing data and materials.

3. We note that you have included the phrase “data not shown” in your manuscript. Unfortunately, this does not meet our data sharing requirements. PLOS does not permit references to inaccessible data. We require that authors provide all relevant data within the paper, Supporting Information files, or in an acceptable, public repository. Please add a citation to support this phrase or upload the data that corresponds with these findings to a stable repository (such as Figshare or Dryad) and provide and URLs, DOIs, or accession numbers that may be used to access these data. Or, if the data are not a core part of the research being presented in your study, we ask that you remove the phrase that refers to these data.

***Author Response:*** Data previously referred to as “data not shown” were not core to the research presented in our study; therefore, we have removed this phrase.

4. Your ethics statement should only appear in the Methods section of your manuscript. If your ethics statement is written in any section besides the Methods, please move it to the Methods section and delete it from any other section. Please ensure that your ethics statement is included in your manuscript, as the ethics statement entered into the online submission form will not be published alongside your manuscript.

***Author Response:*** We have relocated and incorporated our ethics statement into the Methods section, as outlined in the following paragraph.

Page 4, Lines 105-113

This study is a part of an ongoing project “Genetics of Essential Tremor in Turkish Families: Identification of the Causal Variants.” The primary aim of the study is to explore the genetic underpinnings of essential tremor (ET) in familial cases across Turkey. Written (signed) informed consent was obtained from all participants at the time of enrollment. All procedures involving human participants adhered to the ethical standards set by the institutional and national research committees, as well as the 1964 Helsinki Declaration and its later amendments, or comparable ethical guidelines. Ethical approval for the study was obtained from the Institutional Review Boards of both Bilkent University and Ankara University, under the regulations and guidelines issued by the Ministry of Health, Turkey.

5. We are unable to open your Supporting Information file [S2 and S3 File]. Please kindly revise as necessary and re-upload.

***Author Response:*** The Supporting Information files, S2 and S3, contain the Python and R codes used in this study. These codes have been uploaded to a GitHub repository, with the links provided in the “Data Availability Statement” section, in accordance with PLOS ONE’s “Code Sharing” policies. We updated this section as in the following paragraph:

Page 17, Lines 631-636

Data are available in the main text or the supplementary materials. The raw data supporting the conclusions of this article will be made available by the authors without undue reservation. The R Markdown and Python codes used for the analysis are accessible in the GitHub repository (https://github.com/farukustunel/et-longevity-analysis). These codes include scripts for data processing, statistical analysis, and visualization.

Additional Editor Comments:

The manuscript provides valuable insights into the relationship between essential tremor (ET) and longevity. However, there are areas where improvements can enhance the impact of the work.

***Author Response:*** We have revised the entire manuscript and rewritten all sections based on your valuable feedback.

First, the introduction could better contextualize the study by clearly defining the research gaps and how this study specifically addresses them.

***Author Response:*** We revised the introduction part to clearly contextualize the text and addressed the research gaps as in the following paragraph:

Page 3, Lines 87-102

Building on this existing body of research, with contradictory results, further investigation into the relationship between ET and mortality is crucial. In our family-based retrospective study, we aimed to address this gap by comparing mortality risk in ET patients to their family members. We examined the pedigrees of 145 probands and tested the association of ET with longevity in 1,493 subjects. Our findings suggest that ET could indeed be associated with longer life expectancy, offering an intriguing avenue for further research into the underlying mechanisms that might confer this potential longevity advantage. These findings, if confirmed in large-scale longitudinal studies and genetic investigations, could have significant implications for public health, particularly in understanding the complex interplay between ET, aging and survival.

Additionally, the methodology section, while comprehensive, would benefit from further elaboration on data collection procedures, particularly the criteria used to select family members for inclusion.

***Author Response:*** We revised the whole methods section, including the data collection procedures as in the following paragraph:

Page 3-4, Lines 114-151

The clinical assessments were conducted at the Ankara University Medical School Hospital, one of the country’s leading referral centers, renowned for its comprehensive medical expertise. As a government-subsidized institution within Turkey’s universal healthcare system, the hospital serves patients from diverse economic backgrounds. An analysis of the hometowns of the enrolled probands confirmed the diversity of participants, with individuals representing all regions of Türkiye. Moreover, control participants for the study were recruited from within the same families as the probands, ensuring comparable healthcare-seeking behavior between the affected and unaffected groups.

Patients admitted to the Neurology Department of Ankara University Medical School Hospital with the complaint of tremor were invited to participate in the genetic study if they reported having multiple affected and unaffected individuals in their families. Each proband underwent a comprehensive 90-minute baseline medical interview. If patients exhibited neurological symptoms, such as cognitive impairment, bradykinesia, or tremor, they were referred for detailed and follow-up neurological evaluations. We preferentially recruited familial early-onset ET cases, particularly those classified as “pure ET”. Clinical information parameters included: a. type of tremor, b. presence or absence of Parkinson’s disease, c. presence or absence of dystonia, d. response to treatment, e. history of drug use (e.g., lithium), f. substance use (e.g., alcohol or drug addiction), g. exposure to chemicals or toxins. Exclusion criteria were strictly applied to enhance the homogeneity of the study group and minimize confounding variables. We excluded individuals diagnosed with dementia, Parkinson's disease, or any other neurodegenerative disease, as well as those with known vascular, demyelinating, or structural brain lesions (e.g., masses). Additionally, individuals taking medications known to induce action tremor or those with severe hyperthyroidism were also excluded. However, recognizing the association between ET and a heightened risk of Parkinson’s disease, patients with ET who exhibited overlapping parkinsonism features (e.g., bradykinesia, rigidity, and non-motor symptoms) were not excluded from the study [22–24]. Demographic information for each proband was collected during the initial interview. Probands were asked to provide detailed information about all living or deceased first- and second-degree relatives and indicate whether any relatives had tremors.

The accuracy of the pedigree information was critical for genetic analysis, and multiple checks were incorporated into the data collection process to ensure reliability. Recruitment of family members occurred during follow-up appointments, and efforts were made to have as many family members present as possible during these visits. This strategy facilitated the construction of robust pedigree charts, as family members could cross-verify information, minimizing discrepancies. During these appointments, family members collaborated to name and list all living and deceased first- and second-degree relatives, and they jointly reported on the presence of tremors in the family. This cross-referencing process ensured high specificity and accuracy in the familial data collected.

Enhancing the clarity of the statistical methods used would also help readers follow the analysis more easily.

***Author Response:*** To enhance the clarity of the statistical methods we used, we revised the statistical methods section in a more structured and detailed manner, ensuring that each step of our analysis is clearly explained.

Page 7-8, Lines 228-268

Statistical analyses were conducted using R version 4.4.1 [30], while survival analyses were performed in Python version 3.11.5 [31], utilizing the lifelines library version 0.27.8 [32]. To assess the normality of the data, several tests were applied, including the Kolmogorov-Smirnov test, Anderson-Darling test, and Shapiro-Wilk test. In addition, the data distribution was visually examined using normal quantile-quantile (Q-Q) plots and a histogram.

The baseline characteristics of individuals with and without ET were compared using the non-parametric Mann-Whitney U test, which is appropriate for non-normally distributed data. Additionally, the Kruskal-Wallis test was used to compare groups regarding disease severity and age of onset, as this test is suited for unequal sample sizes and non-normal data distributions. To determine specific differences among groups, a post-hoc Dunn test was applied with Holm correction to adjust for multiple comparisons.

For survival analysis, the main outcome of interest was ‘age in years’ for living individuals and ‘age-at-death’ for deceased individuals. Kaplan-Meier survival curves were constructed to estimate and compare survival probabilities between ET+ and ET- groups. Survival comparisons were stratified by cohort and gender to assess whether ET status influenced survival within these subgroups. The log-rank test was performed to statistically compare survival curves, providing indication of whether the differences between groups were significant. To explore the relationship between ET status and overall survival, univariate Cox proportional hazards regression models were employed. This allowed us to assess the correlation between disease status (ET+ or ET-) and survival time. A multivariate Cox regression model was then built to account for potential confounders such as gender, while still assessing the effect of disease status. We used a stepwise model-building approach, sequentially introducing predictor variables and assessing their impact on survival, to ensure the most relevant prognostic factors were included in the final model [33]. Statistical significance was defined by p-value less than 0.05

To provide additional insight into the familial aggregation, we selected 10 families, each consisting of at least 10 individuals, for family-wise comparisons. Given the small sample sizes within these families, selecting the appropriate statistical method was crucial. We first used the Shapiro-Wilk test to assess the normality of age distributions within each family. Visual checks using Q-Q plots and histograms were also conducted for further confirmation. For families where the data significantly deviated from normality, we applied the Mann-Whitney U test to compare age distributions. For those families where the data appeared normally distributed, an unpaired t-test with Welch’s correction was used to account for unequal variances. To complement the frequentist analysis, a Bayesian t-test was also performed, which provided additional insights into the comparison of age distributions between ET+ and ET- individuals within these families. After analyzing the families individually, we combined the data from all 10 families and re-evaluated the overall distribution of ages. The combined data did not follow a normal distribution, as confirmed by the statistical tests and visual inspections. Thus, we employed the Kruskal-Wallis test to perform an overall comparison across families, which allowed us to evaluate differences in age distributions across multiple groups without assuming normality.

Moreover, the discussion could delve deeper into the potential biological mechanisms linking ET to longevity, as this connection remains speculative. Strengthening this section with references to recent studies in the field could offer a more robust interpretation of the findings.

***Author Response:*** In the revised manuscript, we have largely removed speculative sentences and presented potential biological mechanisms in a more cautious and nuanced manner. We have also clarified that these explanations are preliminary and should be considered as hypotheses rather than definitive conclusions. Additionally, we have expanded the discussion to emphasize the need for further research to investigate these potential factors through more rigorous data collection and analysis. Please see following:

Page 15-16, Lines 524-571

Our study suggests an intriguing association between ET and increased longevity, particularly in familial cases. While this association remains speculative, several potential biological mechanisms could explain this connection. It is important to consider that while our study provides novel insights, the underlying biology linking ET to longevity is complex and requires further exploration. One potential biological mechanism is the neuroprotective effect of ET. Although ET is traditionally considered a movement disorder, there is emerging evidence suggesting that certain pathophysiological aspects of the disease may confer protective effects on the nervous system. Recent studies have proposed that the tremor may be related to enhanced neural plasticity, which could play a role in improving neuroprotection and mitigating neurodegeneration in certain brain regions. For example, it has been suggested that the constant neuronal activity associated with tremor may induce adaptive changes that enhance resilience to age-related neurodegeneration [41]. This could potentially explain why individuals with ET might experience slower cognitive decline or improved survival compared to the general population. Another potential mechanism involves the dopaminergic system. While ET is not typically classified as a neurodegenerative disorder like Parkinson’s disease, it has been suggested that some individuals with ET may have compensatory mechanisms involving dopamine or other neurochemicals that confer a neuroprotective effect [[42]](https://www.zotero.org/google-docs/?KVyA4Y). Studies have indicated that the basal ganglia circuitry, which is implicated in motor control and is disrupted in tremor, may also have a role in regulating other neurochemical pathways that influence aging and longevity. For example, dopamine’s role in modulating oxidative stress and inflammation could play a key role in enhancing cellular repair mechanisms and slowing the aging process [43,44].

Our study also raises the possibility that genetic factors related to ET may contribute to longevity. Several studies have identified genetic variations associated with ET that could be linked to pathways involved in aging and longevity. For instance, mutations in genes related to mitochondrial function or oxidative stress response pathways could influence both the development of ET and the lifespan of affected individuals [39,43,45]. Additionally, familial aggregation of ET may suggest the presence of genetic modifiers that influence not only tremor development but also overall health and longevity [46]. These genetic variants may provide protection against age-related diseases or promote better health outcomes as individuals age.

One more plausible explanation for the increased longevity observed in our study could be improved medical compliance among individuals with ET. Chronic conditions such as ET often require regular medical check-ups and adherence to prescribed therapies, which could translate into better management of comorbidities and early detection of other health issues. This is consistent with findings from other studies, which have shown that individuals with chronic diseases tend to seek more frequent medical care and engage in healthier lifestyle practices that contribute to prolonged life. Furthermore, patients with ET may be more likely to adopt preventive measures such as regular exercise or medications, which could contribute to their extended lifespan [[47–49]](https://www.zotero.org/google-docs/?Rjj9kp).

Environmental and lifestyle factors, including diet, physical activity, and alcohol consumption, could also play a role in the longevity of ET patients. Previous research has indicated that moderate alcohol consumption may have neuroprotective effects, potentially alleviating tremor symptoms and improving motor function [[49,50]](https://www.zotero.org/google-docs/?wXnHCx). Exercise, which has been shown to improve cognitive function and overall health, could also have a beneficial effect on individuals with ET, mitigating some of the negative consequences of aging [51,52]. Furthermore, dietary factors such as caloric restriction have been linked to extended lifespan in both animal models and human populations, and it is possible that individuals with ET may adopt diets that reduce inflammation and oxidative stress, both of which contribute to aging [43,45].

Lastly, a thorough proofreading of the manuscript is necessary to correct minor grammatical errors and ensure clarity.

***Author Response:*** We have carefully revised the manuscript, ensuring that we clearly explain our findings and correct any grammatical errors.

**Reviewer 1: Muhammad Zunnurain Hussain**

Dear Dr. Hussain,

We sincerely appreciate the time and effort you have dedicated to evaluating our work. The insightful comments and valuable suggestions we received have greatly contributed to improving the quality of our manuscript.

We have carefully considered all the feedback provided, and we have incorporated most of your suggestions into the revised manuscript. We revised almost the entire text and utilized the track changes feature in Word to clearly highlight all modifications. Additionally, we have prepared a clean version of the manuscript. Below, we provide a point-by-point response to your comments and concerns in blue font. Please note that all page numbers mentioned correspond to the revised manuscript without tracked changes.

With my best regards,

Onur Emre Onat

Small Sample Size: The study’s sample size, though substantial, is still limited. The authors acknowledge that more extensive studies are necessary to confirm the findings and understand the broader implications

***Author Response:*** We appreciate the reviewer’s acknowledgment of the substantial sample size of our study. We fully agree that larger studies would be advantageous for confirming our findings and gaining a deeper understanding of their broader implications. Although our current sample size provides significant insights, we acknowledge its limitations and have addressed this in the revised manuscript. Specifically, we have expanded the discussion to emphasize the need for future research with larger cohorts to validate our results and explore potential variability across different populations. We believe this approach will offer a more comprehensive understanding of the effects of essential tremor on longevity and mortality rates within families. This acknowledgment has been revised and included in the “Discussion” section, as detailed in the following paragraph.

Page 16, Lines 572-580

In conclusion, our study adds to the limited body of literature on ET and mortality by suggesting that ET may be associated with increased lifespan, particularly in male patients and those with late-onset disease. However, our findings should be interpreted with caution due to the study’s limitations, including the reliance on self-reported family histories and the lack of detailed cause-of-death data. Future research should aim to collect more comprehensive biological, genetic, and lifestyle data, as well as to include larger and more diverse cohorts, to validate and extend these findings. Clarifying the mechanisms underlying the relationship between ET and longevity could yield important insights into both ET pathophysiology and the broader biological processes that contribute to healthy aging.

Family-based Study Limitations: By focusing on families, the study may have introduced selection bias. The family-based design might not represent the general population accurately, and the genetic and environmental factors specific to these families may not be generalizable.

***Author Response:*** We reviewed the literature on tremors and longevity, focusing on both retrospective and prospective studies, which commonly utilize a case-control approach. We acknowledge that focusing on families may introduce selection bias, as the genetic and environmental factors observed within these families might not fully represent the broader population. However, in our study, considering that longevity is influenced by numerous factors, we aimed to minimize these variables.

Our cohort consists of families predominantly residing together in small villages, where members live under similar socioeconomic conditions. This homogeneity means their dietary habits, microbial exposures, and living conditions are relatively consistent, although different families may not share these conditions. To address potential biases, we conducted a separate analysis within each family (Fig 4), which yielded results consistent with the analysis where all families were evaluated together (Fig 1).

We believe that familial studies can provide valuable insights into hereditary aspects and intra-family variations in essential tremor. However, we also agree that family-based approaches should be interpreted with caution when generalizing findings to the broader population. We have highlighted this limitation and our approach in the discussion section and emphasized the importance of complementing family-based studies with population-based research to gain a comprehensive understanding of the genetic and environmental influences on essential tremor and its impact on longevity and mortality. Our extended explanations in the “Introduction” and “Discussion” sections of the manuscript are as follows:

Page 3, Lines 87-102

Building on this existing body of research, with contradictory results, further investigation into the relationship between ET and mortality is crucial. There is a clear need for more rigorous, family-based studies to investigate how ET might influence survival rates, particularly by accounting for genetic factors and controlling for confounding variables such as diet and socioeconomic status. Additionally, there is a lack of data exploring gender-specific differences in mortality and how such differences could contribute to our understanding of ET’s impact on longevity. In our comprehensive family-based retrospective study, we aimed to address this gap by comparing mortality risk in ET patients to their family members. By controlling for non-genetic factors, we attempt to provide more conclusive evidence on the relationship between ET and longevity. We examined the pedigrees of 145 probands and tested the association of ET with longevity in 1,493 subjects. Our findings suggest that ET could indeed be associated with longer life expectancy, offering an intriguing avenue for further research into the underlying mechanisms that might confer this potential longevity advantage. These findings, if confirmed in large-scale longitudinal studies and genetic investigations, could have significant implications for public health, particularly in understanding the complex interplay between ET, aging and survival.

Page 13, Lines 453-462

In our own study, we aimed to address some of these gaps by conducting a retrospective family-based analysis comparing ET+ individuals with their ET- relatives. This approach allowed us to control for non-genetic factors, such as diet, lifestyle, and socioeconomic status, which can influence both ET development and longevity. By studying family members from the same households and communities, we sought to minimize these confounding variables. However, this design also introduces the potential for selection bias, as the families we studied may not represent the broader population. The genetic uniqueness of our cohort is underscored by an inbreeding coefficient of 36.5%, calculated from exome sequencing data, indicating a higher degree of genetic relatedness within these families than would be typical in the general population.

Data Collection Methodology: The study relies on historical data provided by participants and their families, which could introduce recall bias and inaccuracies. Many assessments were based on reports rather than direct examinations, which can lead to misclassification of ET status.

***Author Response:*** We thank the reviewer for highlighting a potential source of bias. As discussed in the manuscript, collecting death information from family members represents a limitation. Our attempts to access original death certificates were hindered by complex bureaucratic processes and family members’ reluctance to engage in these procedures.

However, we want to emphasize the robustness of our data collection methods. Family member recruitments were conducted during appointments where all members were present, ensuring coordinated responses. Pedigree drawings, naming and listing of living and deceased first- and second-degree relatives, and reports of tremors were completed in the presence of multiple family members, with data cross-checked and verified to achieve high specificity. The revised text reads as follows:

Page 5, Lines 143-151

The accuracy of the pedigree information was critical for genetic analysis, and multiple checks were incorporated into the data collection process to ensure reliability. Recruitment of family members occurred during follow-up appointments, and efforts were made to have as many family members present as possible during these visits. This strategy facilitated the construction of robust pedigree charts, as family members could cross-verify information, minimizing discrepancies. During these appointments, family members collaborated to name and list all living and deceased first- and second-degree relatives, and they jointly reported on the presence of tremors in the family. This cross-referencing process ensured high specificity and accuracy in the familial data collected.

While a longitudinal prospective study would be ideal to address this limitation, such studies are challenging given the retrospective nature of our research and the fact that many patients are deceased. As an alternative, we tested our data collection methodology by comparing ET patients and non-ET family members across 10 well-characterized multigenerational families with both affected and unaffected individuals. We confirmed our observations in all families, although significant changes were only evident in 3 of the 10 families due to the small sample size. Additionally, we compared the survival status of ET patients with and without clinical assessment and found no significant difference in age at death between the two groups (Figure 2E). The revised text reads as follows:

Page 14, Lines 498-508

Indeed, our study does have limitations that should be carefully considered. First, 35.6% of ET+ cases and 44.3% of ET- individuals were deceased family members, and 22 individuals had died after the baseline evaluation. For many of these individuals, assessments were based on family-reported histories rather than direct clinical evaluations, which introduces the potential for recall bias and misclassification of ET status. To mitigate this, we compared survival data between ET patients with and without clinical assessments, finding a significant difference in age at death between the two groups (U = 2.99 × 103, p = 2 × 10-3) (Fig 2E). Furthermore, we examined 10 phenotypically well-characterized multigenerational families, finding that in 3 of the 10 families, ET patients lived longer than their non-ET relatives. This finding suggests that genetic factors contributing to ET may also play a role in influencing longevity [40].

Lack of Detailed Cause of Death Data: For many deceased individuals, the exact cause of death was not known and was broadly classified as "natural death," which could obscure specific causes that might influence longevity.

***Author Response:*** We appreciate the reviewer’s concern regarding the lack of detailed cause of death data in our study. We agree that the broad classification of "natural death" may limit our ability to identify specific causes that could significantly impact longevity and mortality analysis. As previously mentioned, our efforts to access original death certificates were unsuccessful due to complex bureaucratic processes and family members' reluctance to engage in these procedures.

In our study, the most reported cause was "natural death," particularly for earlier generations in the control group. Due to the lack of detailed clinical examinations in rural settings during those times, exact causes of death were often unknown and categorized as natural death. However, causes such as heart attack, stroke, cancer, various accidents, respiratory diseases, and infectious diseases were also reported. We have included this information in the follow-up evaluation section. To address this limitation, we focused on individuals aged 60 and over from our study group and repeated the analysis. This refined analysis revealed a significant increase in survival time for ET patients compared to non-ET family members (HR=2.17, 95% CI=1.73-2.73, P < 0.0001) (Fig. 3A). The revised text reads:

Page 14, Lines 508-514

One of the major limitations of our study is the lack of detailed cause of death data for many individuals. Many deceased participants were broadly classified as having died of "natural causes," which limits our ability to explore specific mortality factors that might influence the association between ET and longevity. Access to more precise cause-of-death data, such as death certificates or autopsy reports, would provide valuable insights into whether specific comorbidities or conditions, such as cardiovascular disease or neurodegenerative disorders, are common in ET patients compared to the general population.

Page 16, Lines 572-580

In conclusion, our study adds to the limited body of literature on ET and mortality by suggesting that ET may be associated with increased lifespan, particularly in male patients and those with late-onset disease. However, our findings should be interpreted with caution due to the study’s limitations, including the reliance on self-reported family histories and the lack of detailed cause-of-death data. Future research should aim to collect more comprehensive biological, genetic, and lifestyle data, as well as to include larger and more diverse cohorts, to validate and extend these findings. Clarifying the mechanisms underlying the relationship between ET and longevity could yield important insights into both ET pathophysiology and the broader biological processes that contribute to healthy aging.

Survival Analysis: The paper mentions potential biases in survival analysis, such as the possibility that individuals who lived longer were more likely to develop ET, rather than ET contributing to longer life. This reverse causation could affect the interpretation of results.

***Author Response:*** Recent mortality studies have reported contrasting findings in patients with ET, showing significantly higher or lower mortality rates. However, nearly all studies find the most convincing association with longevity in cases of late-onset tremors in elderly populations. We agree with the reviewer’s comment regarding potential biases in the survival analysis, particularly the concern about reverse causation. We have already discussed the possibility that individuals who lived longer may have been more likely to develop essential tremor (ET), which could confound our results by making it appear that ET contributes to increased longevity.

To address this bias, we evaluated the effect of age of onset on age distributions in our study. Our results showed that the most significant increase in longevity was observed in the late-onset group, but early- and intermediate-onset ET cases also lived significantly longer than the control group. The Kruskal-Wallis test revealed a statistically significant increase in median ages as disease onset decreased (χ² = 145.65, df = 2, p = 2.36 × 10⁻³²). The median ages in early, intermediate, and late-onset ET cases were 56.5, 64, and 79, respectively. Furthermore, all pairwise comparisons for age of onset were statistically significant (early-intermediate, p = 4.28 × 10⁻³; early-late, p = 1.38 × 10⁻²⁷; intermediate-late, p = 5.18 × 10⁻²³) (Figs. 1C-D).

To address the possibility of reverse causation, where individuals who lived longer may have been more likely to develop ET, we have expanded the discussion in the revised manuscript to explore this further. The revised text reads as:

Page 14-15, Lines 514-520

Another potential source of bias in our study is the question of whether control individuals who died before the age of 50 would have been diagnosed with ET if they had lived longer. While we cannot entirely rule out this possibility, our analysis of individuals aged 60 and older still demonstrated a significant increase in survival times for ET patients (Log-rank p = 5.29 × 10-16 (Fig 3B)), suggesting that the longevity benefit associated with ET is robust even among older individuals.

Confounding Variables: Although the study attempts to control for certain variables, there are many potential confounders (e.g., lifestyle factors, comorbidities) that were not accounted for, which could influence the association between ET and longevity.

***Author Response:*** As previously mentioned, our study design focused on families to minimize the impact of external variables. Our cohort consists of families predominantly residing in small villages, where members share similar socioeconomic conditions. This homogeneity extends to dietary habits, microbial exposures, and living conditions. Unfortunately, we were unable to obtain detailed clinical data on all potential comorbidities, as these variables were not measured or recorded during the original data collection. In the revised manuscript, we have expanded the discussion to explicitly acknowledge this limitation and its potential impact on our findings. The revised text reads as follows:

Page 15, Lines 520-523

Additionally, our study did not account for certain lifestyle factors, such as diet, physical activity, and alcohol consumption, which may influence both ET development and longevity. Further studies that incorporate detailed data on these factors would help to clarify their potential impact.

Page 16, Lines 576-578

Future research should aim to collect more comprehensive biological, genetic, and lifestyle data, as well as to include larger and more diverse cohorts, to validate and extend these findings.

Page 16, Lines 563-571

Environmental and lifestyle factors, including diet, physical activity, and alcohol consumption, could also play a role in the longevity of ET patients. Previous research has indicated that moderate alcohol consumption may have neuroprotective effects, potentially alleviating tremor symptoms and improving motor function [[49,50]](https://www.zotero.org/google-docs/?wXnHCx). Exercise, which has been shown to improve cognitive function and overall health, could also have a beneficial effect on individuals with ET, mitigating some of the negative consequences of aging [51,52]. Furthermore, dietary factors such as caloric restriction have been linked to extended lifespan in both animal models and human populations, and it is possible that individuals with ET may adopt diets that reduce inflammation and oxidative stress, both of which contribute to aging [43,45].

Gender Differences: The study finds gender-specific differences in survival rates, suggesting that males with ET might have a survival advantage. However, the underlying reasons for this gender disparity are not explored in depth, leaving questions about the mechanisms driving these differences.

***Author Response:*** In the revised manuscript, we have expanded the discussion to emphasize the importance of understanding gender differences in essential tremor (ET). We highlight that previous studies have observed variations in ET presentation and prevalence between genders, such as the higher prevalence of head tremor in women and the predominance of ET in males during childhood. Future research should investigate the mechanisms underlying these gender-specific outcomes to better understand their implications for ET-related survival rates. The revised text reads as follows:

Pages 13-14, Lines 463-488

Our findings provide important insights into the relationship between ET and mortality. We found that the median age of death for the ET+ group was significantly higher than for ET- individuals (80 years vs. 70 years), suggesting that ET might be associated with increased longevity. This was further supported by survival analysis, which revealed a statistically significant higher risk of mortality in ET- individuals compared to those with ET. Interestingly, we observed notable gender-specific differences in survival. Males had a log-rank p-value of 1.38 × 10-16 (HR = 1.51, CI95% = 1.08-2.13), while females had a log-rank p-value of 1.0 × 10-9 (HR = 1.37, CI95% = 0.98-1.93), indicating that the longevity benefits of ET were more pronounced in men. These findings align with prior studies that have suggested gender differences in ET expression, such as the higher prevalence of head tremor in women and the predominance of ET in males during childhood. The potential gender disparity in ET-related longevity raises important questions about the biological mechanisms that may underlie these differences. It is well-documented that men and women experience different patterns of disease progression and comorbidity risk in many neurological and age-related conditions, and ET may be no exception. Further research is needed to investigate the specific factors that might contribute to the greater longevity observed in male ET patients, including genetic, hormonal, and lifestyle differences [35,36]. When comparing our findings to national mortality data, we found that the median age of death in our cohort (74 years; males: 71, females: 77) was in line with the average life expectancy of the Turkish population in 2019, which was 78 years (males: 76, females: 81) [37]. However, when we focused on ET patients, their median age of death was 70 years (males: 68, females: 71), compared to 64 years (males: 64, females: 65) for their healthy relatives. While this suggests that ET patients in our study tended to live longer than their unaffected family members, we also found that the average year of death for deceased individuals in the ET- group was 2006 (1992-2021). This suggests that the time period during which deaths occurred may influence the observed differences in longevity, as life expectancy in Turkey has increased over the past several decades.

Speculative Interpretations: The authors present several speculative explanations for their findings (e.g., dietary habits, medical compliance, alcohol consumption, exercise benefits), but these are not backed by solid biological data within the study, requiring further investigation.

***Author Response:*** We agree with the reviewer that there is a lack of a clear biological explanation for our findings, which align with previously reported observations. In the revised manuscript, we have largely removed speculative interpretations and presented possible explanations in a more cautious and nuanced manner. We have also clarified that these explanations are preliminary and should be considered as hypotheses rather than definitive conclusions. Additionally, we have expanded the discussion to emphasize the need for further research to investigate these potential factors through more rigorous data collection and analysis. The revised text reads as follows:

Pages 15-16, Lines 524-580

Our study suggests an intriguing association between ET and increased longevity, particularly in familial cases. While this association remains speculative, several potential biological mechanisms could explain this connection. It is important to consider that while our study provides novel insights, the underlying biology linking ET to longevity is complex and requires further exploration. One potential biological mechanism is the neuroprotective effect of ET. Although ET is traditionally considered a movement disorder, there is emerging evidence suggesting that certain pathophysiological aspects of the disease may confer protective effects on the nervous system. Recent studies have proposed that the tremor may be related to enhanced neural plasticity, which could play a role in improving neuroprotection and mitigating neurodegeneration in certain brain regions. For example, it has been suggested that the constant neuronal activity associated with tremor may induce adaptive changes that enhance resilience to age-related neurodegeneration [41]. This could potentially explain why individuals with ET might experience slower cognitive decline or improved survival compared to the general population. Another potential mechanism involves the dopaminergic system. While ET is not typically classified as a neurodegenerative disorder like Parkinson’s disease, it has been suggested that some individuals with ET may have compensatory mechanisms involving dopamine or other neurochemicals that confer a neuroprotective effect [42]. Studies have indicated that the basal ganglia circuitry, which is implicated in motor control and is disrupted in tremor, may also have a role in regulating other neurochemical pathways that influence aging and longevity. For example, dopamine’s role in modulating oxidative stress and inflammation could play a key role in enhancing cellular repair mechanisms and slowing the aging process [43,44].

Our study also raises the possibility that genetic factors related to ET may contribute to longevity. Several studies have identified genetic variations associated with ET that could be linked to pathways involved in aging and longevity. For instance, mutations in genes related to mitochondrial function or oxidative stress response pathways could influence both the development of ET and the lifespan of affected individuals [39,43,45]. Additionally, familial aggregation of ET may suggest the presence of genetic modifiers that influence not only tremor development but also overall health and longevity [46]. These genetic variants may provide protection against age-related diseases or promote better health outcomes as individuals age.

One more plausible explanation for the increased longevity observed in our study could be improved medical compliance among individuals with ET. Chronic conditions such as ET often require regular medical check-ups and adherence to prescribed therapies, which could translate into better management of comorbidities and early detection of other health issues. This is consistent with findings from other studies, which have shown that individuals with chronic diseases tend to seek more frequent medical care and engage in healthier lifestyle practices that contribute to prolonged life. Furthermore, patients with ET may be more likely to adopt preventive measures such as regular exercise or medications, which could contribute to their extended lifespan [47–49].

Environmental and lifestyle factors, including diet, physical activity, and alcohol consumption, could also play a role in the longevity of ET patients. Previous research has indicated that moderate alcohol consumption may have neuroprotective effects, potentially alleviating tremor symptoms and improving motor function [49,50]. Exercise, which has been shown to improve cognitive function and overall health, could also have a beneficial effect on individuals with ET, mitigating some of the negative consequences of aging [51,52]. Furthermore, dietary factors such as caloric restriction have been linked to extended lifespan in both animal models and human populations, and it is possible that individuals with ET may adopt diets that reduce inflammation and oxidative stress, both of which contribute to aging [43,45].

In conclusion, our study adds to the limited body of literature on ET and mortality by suggesting that ET may be associated with increased lifespan, particularly in male patients and those with late-onset disease. However, our findings should be interpreted with caution due to the study’s limitations, including the reliance on self-reported family histories and the lack of detailed cause-of-death data. Future research should aim to collect more comprehensive biological, genetic, and lifestyle data, as well as to include larger and more diverse cohorts, to validate and extend these findings. Clarifying the mechanisms underlying the relationship between ET and longevity could yield important insights into both ET pathophysiology and the broader biological processes that contribute to healthy aging.

Potential Misclassification: The study includes ET patients with overlapping parkinsonism features, which could complicate the classification and analysis. The exclusion criteria were stringent, but the presence of non-motor symptoms and other conditions might still affect the results.

***Author Response:*** In the revised manuscript, we have detailed the steps taken to mitigate misclassification, including the stringent exclusion criteria applied to our cohort. We also recognize that despite these measures, some degree of overlap between ET and parkinsonism could still affect our findings. We have expanded our discussion to address this limitation and its potential impact on the study’s conclusions. Additionally, we recommend that future research incorporate more precise diagnostic criteria and consider additional diagnostic tools to better differentiate between ET and parkinsonism-related conditions. The revised text reads as follows:

Page 4, Lines 131-139

Exclusion criteria were strictly applied to enhance the homogeneity of the study group and minimize confounding variables. We excluded individuals diagnosed with dementia, Parkinson's disease, or any other neurodegenerative disease, as well as those with known vascular, demyelinating, or structural brain lesions (e.g., masses). Additionally, individuals taking medications known to induce action tremor or those with severe hyperthyroidism were also excluded. However, recognizing the association between ET and a heightened risk of Parkinson’s disease, patients with ET who exhibited overlapping parkinsonism features (e.g., bradykinesia, rigidity, and non-motor symptoms) were not excluded from the study [22–24].

Page 10, Lines 334-337

On the other hand, the presence of parkinsonism (ET+PD+) did not significantly affect survival patterns within the deceased cohort (*U* = 2.838, *p* = 0.05), suggesting that the overlap of ET and Parkinson’s disease did not contribute to a noticeable difference in longevity. (**Fig 2F**).
